# Supplementary material for: Intrinsic structural and electronic properties of the Buffer Layer on Silicon Carbide unraveled by Density Functional Theory
Source: Sci Rep. 2018 Aug 30;8:13097. doi: 10.1038/s41598-018-31490-7 (PMC6117312; doi:10.1038/s41598-018-31490-7)
Supplement: Supplementary file 1 — Supporting Information [file 41598_2018_31490_MOESM1_ESM.pdf]

Intrinsic structural and electronic properties of the Buffer Layer on Silicon Carbide unraveled by Density Functional Theory

Tommaso Cavallucci and Valentina Tozzini

NEST- Scuola Normale Superiore and Istituto Nanoscienze, Cnr, Piazza San Silvestro 12, 56127 Pisa, Italy

Supporting information

S.1. Distributions of bonds

As mentioned in the main text, the covalent bonds distribution can be evaluated in different ways (i) by evaluation of the z coordinate (ii) by selecting the Si-C minimum distance for each C atom in the BL and (iii) evaluating the charge density value in a point midway between each C atom in the BL and the nearest Si atom (see Fig S.1 b). Fig S.1 (a) compares the methods (ii) and (iii) showing the distribution of Si-C minimal distances and the values of charge density in the midway point. The two sets of distribution (three for each method, for the three models) both display three separate populations, corresponding to the bound atoms (small Si-C distance, high density), the unbound atoms (intermediate distance and density) and crests atoms (large distance, small density). The populations selected with the two methods coincide and are represented in colors in Fig S.2, (a-c).

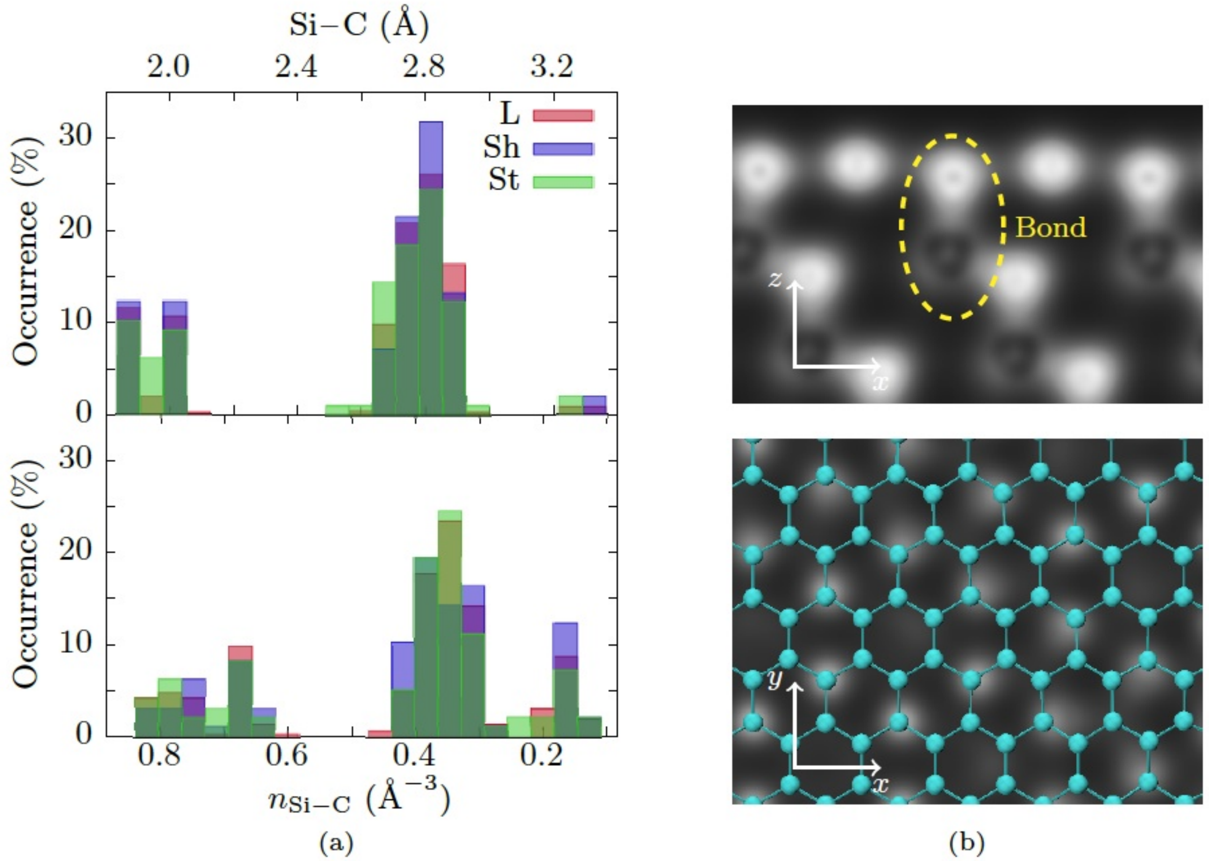

Fig S.1 (a) Distributions of the minimum distances between C atoms of the BL and Si atoms of the substrate (top) and of the charge density value evaluated in the middle points of the Si-C distance from a C of the buffer and the nearest Si (the latter shown with inverted abscissa, to better compare with the fomer). (b) Total electronic charge density evaluated on a vertical plane cutting some Si-C bonds (indicated) and on an horizontal plane located between the BL and the substrate.

The comparison of methods (ii)-(iii) and (i) is reported in Fig S.2, (d)-(e). It can be seen that the population of bound and unbound atoms are not very well separable on the basis of the z coordinate, because their tail superimpose. The bonds spatial distribution is visible also in Fig S.3 as they appear as bright spots when the charge density is plotted on a plane between the BL and the substrate. In Fig S.3, also visible (less bright) is the aromatic π system of the "benzene" rings (corresponding to "unbound" atoms in dark grey in Fig S.2). As explained in the text, the rings are separated by bound atoms.

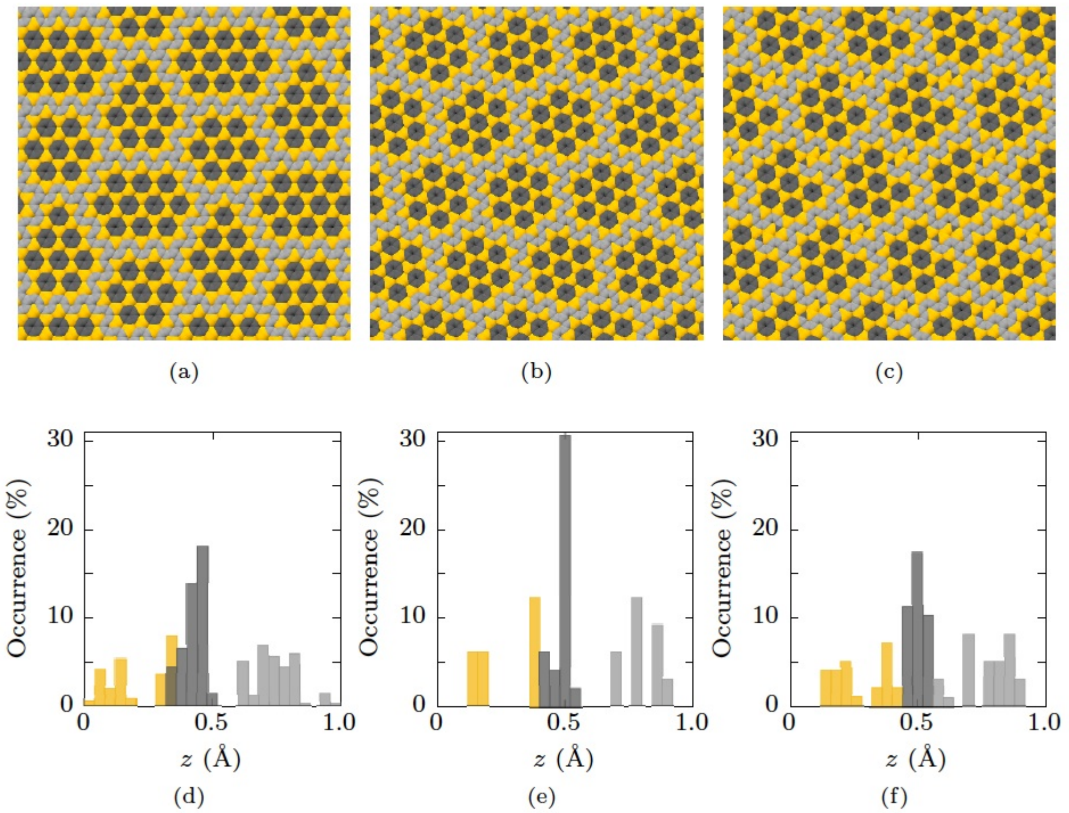

Fig S.2 Space distribution of bound (yellow), unbound (dark grey) and crests atoms (light grey), in L (a), Sh (b) and St (c) models, as selected with the method (ii). (d-f) report the height distribution of the three classes of atoms, colored as in (a-c).

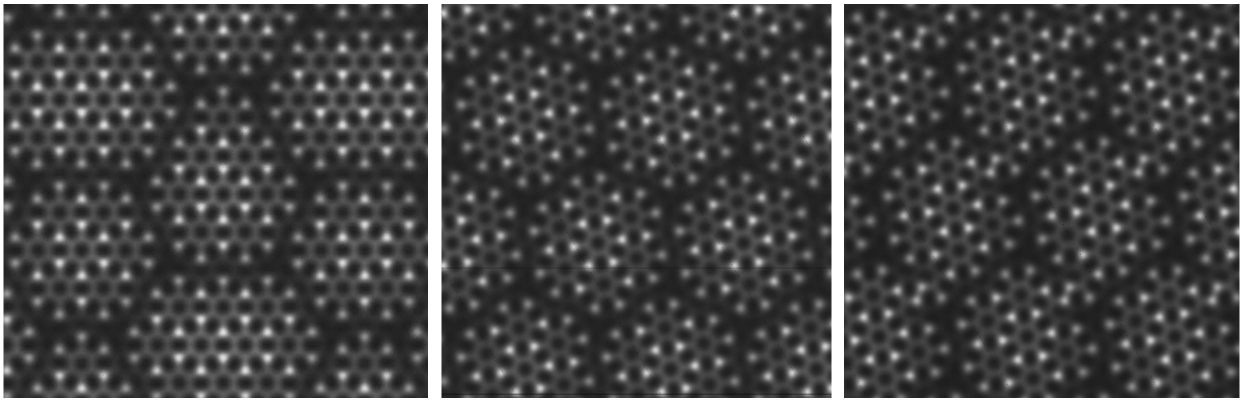

(a) (b) (c)

Fig S.3 Charge density distribution plotted on a plane located between the BL and the substrate, as in Fig S.1 (b) (upper), for the three models (a)=L, (b)=Sh, (c)=St.

S.2. Binding energy

An alternative to the formation energy Ef to evaluate the relative stability of the models is the binding energy, e.g. the energy of the system evaluated with respect to the graphene sheet separated from the substrate

$$E_b = E_{opt} - (E_{sub} + E_{gr})$$

being E_{opt}, E_{sub} and E_{gr} the energy of the systems and of their substrate and graphene isolated components respectively evaluated in the same cell. E_b accounts both for the chemical energy included in the covalent

bonds between the buffer and the substrate and of their structural readjustment due to binding. This quantity (and its value per unit surface, per Si atom, per C atom and per bond is reported in Table S.1.

For S models the estimated dissociation energy of the buffer to the separated graphene is ~0.7eV (little larger for Sh) per Si-C covalent bond, which is little less than what expected for an average value for a covalent bond. Conversely the value for L model is considerably larger, indicating that the sheet is more difficult to detach in the L model than in S. We remark, however, that this is just a very rough evaluation of the binding energy, especially because the reference system is not the flat graphene, but a laterally compressed graphene sheet. Therefore the reference systems in the two cases are different being the lateral compression different.

	E_b (eV)	N_{Si}	N_C	N_b	N_b/N_C	N_C/S (nm^{-2})	E_b/N_{Si} (eV)	E_b/N_C (eV)	E_b/N_b (eV)
**L**	-199.667	108	338	83	24.6%	38.306	-1.849	-0.5907	-2.406
**Sh**	-16.870	31	98	24	24.5%	38.654	-0.544	-0.1721	-0.703
**St**	-16.577	31	98	25	25.5%	38.654	-0.535	-0.1692	-0.663

Table S.1 Binding energies E_b of the three model systems as defined in the main text, and normalized to the to the number of Si surface atoms, of C buffer atoms and to the number of Si-C bonds, also reported. The average surface density of the buffer atoms, N_C/S is also reported.
